# Variability in Serious Safety Event Classification among Children’s Hospitals: A Measure for Comparison?

**DOI:** 10.1097/pq9.0000000000000613

**Published:** 2022-10-18

**Authors:** Amy Poppy, Sonja I Ziniel, Daniel Hyman

**Affiliations:** From the * Children’s Hospital Colorado, Division of Quality and Patient Safety, Aurora, Colorado; † Children’s Hospital Colorado Division of Quality and Patient Safety and University of Colorado School of Medicine, Department of Pediatrics, Section of Pediatric Hospital Medicine, Aurora, Colorado; ‡ Children’s Hospital of Philadelphia Center for Healthcare Quality and Analytics and Perelman School of Medicine, Department of Pediatrics and the Leonard Davis Institute, University of Pennsylvania Philadelphia, Pennsylvania.

## Abstract

**Introduction:**

Hospitals have no standard for measuring comparative rates of serious safety events (SSE). A pediatric hospital safety collaborative has used a common definition and measurement system to classify SSE and calculate a serious safety event rate. An opportunity exists to evaluate the use of this measurement system.

**Methods:**

A web-based survey utilizing 7 case vignettes was sent to 132 network hospitals to assess agreement in classifying the vignettes as SSEs. Respondents classified the vignettes according to the taxonomy used at their respective organizations for deviations and SSE classification.

**Results:**

Of the 82 respondents, 67 (82%) utilized the same SSE classification system. Respondents did not assess deviations for 2 of the 7 vignettes, which had clear deviations. Of the remaining 5 vignettes, 3 had a substantial agreement of deviation (>85%, Gwet’s AC ≥ 0.68), and 2 had fair agreement (<70%, Gwet’s AC ≤ 0.39). Four of the 7 vignettes had a substantial agreement on SSE classification (>80%; Gwet’s AC ≥ 0.80), and 3 had slight to moderate agreement (<70%, Gwet’s AC ≤ 0.78).

**Conclusions:**

Results demonstrated agreement and variability in determining deviation and SSE classification in the 7 vignettes. Although the SSE methodology and metric used by participant pediatric hospitals yields generally similar review results, one must be cautious in using the SSE rate to compare patient safety outcomes across different hospitals.

## INTRODUCTION

The 1999 Institute of Medicine report, *To Err Is Human*, galvanized commitment by the healthcare industry and its key stakeholders to work systematically to improve patient safety and demonstrate a reduction in the rates of preventable harm. Developing standards and accurate measures for patient safety improvement have been an evolving process.^1–6^ While there are metrics for specific types of hospital-acquired harms (eg, hospital-acquired infections, adverse drug events, pressure injuries)^7–9^ and others proposed,^2,4,10^ there is not an agreed-upon single standard, accurate, validated measure to assess overall patient safety improvement.^1,3,4–6,11–13^ In this context, the evolution and spread of many types of public reporting of quality and safety metrics has resulted in a limited benefit to people seeking either to choose among care providers or to evaluate improvements in safety in individual hospitals, health systems, regions or countries.^2,6,14–16^

Two organizations of Children’s Hospitals in the United States and Canada are working collaboratively to eliminate serious harm across all Children’s Hospitals. The first is Children’s Hospitals’ Solutions for Patient Safety (SPS), a network of over 145 organizations.^17^ The second is the Child Health Patient Safety Organization (Child Health PSO), the only federally registered Patient Safety Organization focused solely on Children’s Hospitals.^18^ Through these networks, member hospitals receive resources and training on identifying and measuring serious harm and performing root cause analyses. Hospital leaders and staff are trained on strategies for improving their organization’s culture of safety^19^ and participate in ongoing sharing and educational opportunities regarding harm reduction through cultural change and system reliability.^20^ One of SPS’s fundamental principles is that “we do not compete on safety”^19^; the network does, however, share comparative data to better understand performance differences across hospitals and learn from organizational successes. Children’s hospitals have successfully reduced rates of hospital-acquired harm through these collaborative learning networks.^19–22^

Children’s Hospitals participating in SPS and the Child Health Patient Safety Organization received specific training on the definition and classification of serious safety events (SSE). SSEs are incidents where a deviation from generally accepted practice standards (GAPS) results in significant temporary or permanent harm or the death of a patient.^11,12,23^ The methodology utilized in these two networks was the Safety Event Classification (SEC) system developed by Healthcare Performance Improvement (HPI), LLC.^23^ The SEC provides a taxonomy and definitions for SSE evaluation which hospitals use to measure performance in reducing patient harm.^23^ The HPI SEC comprises five levels of harm for SSEs, four levels of harm for precursor safety events, and three levels of harm for near-miss events.^23^ SPS network hospitals received this standard taxonomy; however, some hospitals have, over time, adapted their use of these definitions and scales or adopted other SSE classification systems. Regardless of the level of harm scale used, children’s hospitals within SPS have demonstrated reductions in SSEs measured using the Serious Safety Event Rate (SSER) through comprehensive patient safety programs.^24,25^

The SSER is the rate of SSEs per 100,000 adjusted patient days per year and is expressed as a rolling 12-month average at the network^20,23^ and individual hospital levels.^24,25^ HPI has guided hospitals regarding “significant harm.” Still, several factors may contribute to variation in the measurement and reporting of the SSER across different hospitals and even over time in a single hospital. As a result, there is potentially significant variability in evaluating adverse events, assessing what constitutes a deviation from GAPS, and interpreting HPI’s severity classification language.

This article reports the results of a survey designed to assess the variability in SSE classification across SPS hospitals using a set of example case vignettes that highlight challenges in the determination of both deviations from GAPS and in the assessment of harm severity.

## METHODS

The authors developed 7 vignettes from de-identified generalized incidents representative of potential SSE cases where SSE review teams need to determine if a deviation from GAPS occurred and/or the severity of harm that occurred (Table 1). We designed the vignettes to reflect the cases that, over the years, we found challenging to agree on SSE classification based on either the deviation or the severity of harm. Therefore, vignettes included a proposed deviation with harm and the impact of the harm.

**Table 1.** Seven Vignettes Used in Survey (Details in Vignettes Provided to Respondents Have Been Removed or Generalized to Anonymize Vignettes)

	*Lumbar Puncture Specimen*
1	Specimen from Lumbar Puncture (LP) is unintentionally mis-sent through pneumatic tube system and when found it is not able to be evaluated.
	Impact of Harm:Repeat LP under general anesthesia.
	*Outpatient Overdose*
	Overdose of medication from ambulatory clinic.
2	Impact of Harm:Altered mental status in clinic, admitted to inpatient unit for monitoring of altered mental status, gait instability monitoring, continuous pulse ox, and strict I/O’s. Discharge to home 24 hours later at baseline condition.
	*Misdosage of ACE Inhibitor*
3	Initiation of ACE inhibitor in critically ill neonate. Started at 3x the typical starting dose for condition, but within the dosing range in formulary. Infant developed hypotension and decreased urine output overnight. Potassium supplement not discontinued after oliguria noted.
	Impact of Harm:V-tach arrest with hyperkalemia. Required ECMO for 24 hours then recovered.
	*Surgical Abdomen Requiring Laparotomy*
4	Teen with presentation of nausea and intermittent abdominal pain evaluated in ED. Given ondansetron in triage and improved with monitoring. Relevant physical examination not documented prior to discharge.
	Impact of Harm:Admitted at outside hospital with perforated appendicitis, complex course with several surgeries and extended admission.
	*Intrathecal Overdose*
5	Overdose of intrathecal medication (10-fold) during an operative procedure with respiratory depression.
	Impact of Harm:Admitted to PICU and remained intubated postoperatively for 24 hours instead of being extubated and transferred to inpatient surgical unit after procedure as planned.
	*Foreign Body – Delayed Identification*
6	Multiple visits for recurring symptoms including a hospitalization. Foreign body ingestion event had been observed before first of series of visits.
	Impact of Harm:Required several procedures to address impact of prolonged foreign body exposure (vs. one procedure if identified promptly).
	*Syringe Pump Failure*
7	Syringe pump was infusing epinephrine in a fragile patient s/p cardiac surgery. The pump failed during the infusion and was found not to have documentation of maintenance following prior reports of errors/failures.
	Impact of Harm:Hypotension to 50s/30s requiring fluid bolus, restart of epinephrine and albumin. NoCompressions done. The patient fully recovered.

The vignettes were distributed via a web-based survey titled “Solutions for Patient Safety: Survey on SSEs” in REDCap (NIH/NCATS Colorado CTSA Grant Number UL1 TR002535).^26^ An invitation to participate was emailed to the identified quality leaders at the 132 SPS member hospitals at the time of survey dissemination in October 2019. The email contained the survey link and a Word document of the survey questions to allow respondents to gather information and discuss the cases for classification purposes with their SSE review team as they would normally do. Hospitals with more than one identified quality leader were instructed to discuss the cases as a group and submit one response per hospital. Non-responding hospitals received four reminders to complete the survey over 6 weeks, and the survey closed after seven weeks. The University of Colorado School of Medicine Institutional Review Board classified this activity as exempt (COMIRB 19-2201).

The survey had two sections: the first included questions about the characteristics of the pediatric hospitals relating to their history with SPS, the SSE taxonomy system used, characteristics of their SSE review team, and its structures and processes (results not presented). The second section comprised the 7 case vignettes to be considered and scored by the respondents using the SSE taxonomy system in their hospitals. Respondents completed a series of questions for each vignette: first, if their hospital SSE review team would consider the care described to be a deviation from GAPS; second, if their hospital SSE review team would consider this deviation an SSE based on the severity of harm, and third, using the SSE taxonomy the respondents indicated at the beginning of the survey, what level of harm they assigned for events they would classify as SSEs (level of harm results not presented). All survey questions were optional, allowing respondents to answer at their discretion. The authors created the survey with input from patient safety team members at one pediatric hospital and the Clinical Steering Team members from SPS. Five respondents from different institutions field-tested an initial version before the survey was finalized and disseminated.

Gwet’s agreement coefficient (Gwet’s AC) was used to assess agreement among institutions regarding the presence of a deviation from GAPS and the classification of cases as an SSE.^27^ Gwet’s AC is a chance-adjusted agreement coefficient for categorical variables and multiple raters with ranges from 0 to 1. Values of <0.21 indicate slight agreement, 0.21–0.40 indicate fair agreement, 0.41–0.60 indicate moderate agreement, 0.61–0.80 indicate substantial agreement, and values of 0.80–1.0 indicate almost perfect agreement.^28^ Analyses were performed using Stata/SE 17.0 (StataCorp, College Station, Tex.).

## RESULTS

Of the 132 invitations sent, one email was invalid, resulting in 131 invitations to participate. Eighty-two hospitals submitted surveys with a response rate of 62.6% (AAPOR RR1).^29^ Table 2 shows the results for the reported SSE taxonomy utilized. Most (82%) hospitals utilize the HPI SSE 1–5 taxonomy, while 7% reported using an SSE 1–4 taxonomy often referred to as the “Ohio modification.” Nine percent of hospitals reported using other classification systems from the Institute for Healthcare Improvement or the Agency for Healthcare Research and Quality, the Safety Assessment Code Matrix, the Pennsylvania Patient Safety Authority Harm Score Taxonomy, or the National Coordinating Council for Medication Error Reporting and Prevention (NCC MERP) Severity Index Coding. Two hospitals reported not using an SSE classification taxonomy to measure or assess events.

**Table 2.** Serious Safety Event (SSE) Taxonomies Used by Respondent Hospitals

Serious Safety Event (SSE) Taxonomy System Used (n = 82)	N (%)
Healthcare Performance Consulting (HPI) SSE 1–5	67(82)
Ohio modification SSE 1–4	6 (7)
Other	7(9)
Don’t use an SSE classification to measure or assess events	2 (2)

**Fig. 1.**
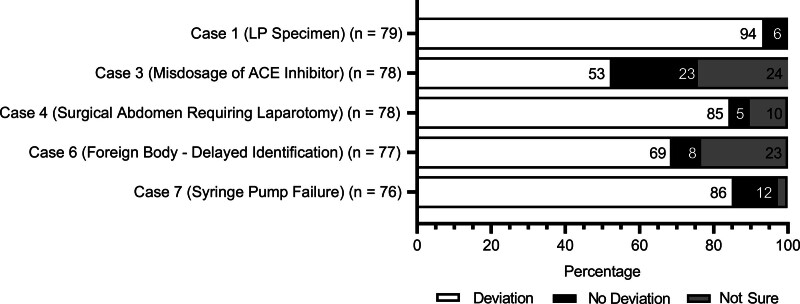
Deviation classification of vignettes.

The hospitals not using an SSE classification taxonomy were excluded from the vignette assessment component of the survey resulting in the opportunity for 80 respondents to assess the vignettes. Figure 1 shows the results of these hospitals’ assessments of a deviation from GAPS. The number of responses varies for each vignette as the survey questions were optional. Vignettes 2 and 5 (medication overdoses) contained clear deviations from GAPS; thus, respondents did not assess for a deviation. There was moderate agreement among responses in the remaining five vignettes (Mean Gwet’s AC = 0.55). Vignette 3 (misdosage of ACE inhibitor) prompted the greatest dissimilarity in responses, with 53% of respondents categorizing the care as a deviation (n = 41 of 78), 23% of respondents determining no deviation from GAPS occurred (n = 18 of 78), and 24% indicating uncertainty (n = 19 of 78) (Gwet’s AC = 0.11). Vignette 6 (foreign body) produced similar variability with 69% determining a deviation from GAPS occurred (n = 53 of 77), 8% indicating no deviation (n = 6 of 77), and 24% remaining uncertain (n = 18 of 77) (Gwet’s AC = 0.39). Vignettes 1 (LP specimen), 4 (surgical abdomen), and 7 (syringe pump failure) demonstrated substantial or almost perfect levels of agreement, with at least 85% indicating a deviation from GAPS occurred (Gwet’s AC = 0.86, n = 74 of 79; Gwet’s AC = 0.68, n = 66 of 78; and Gwet’s AC = 0.71, n = 65 of 76, respectively).

**Fig. 2.**
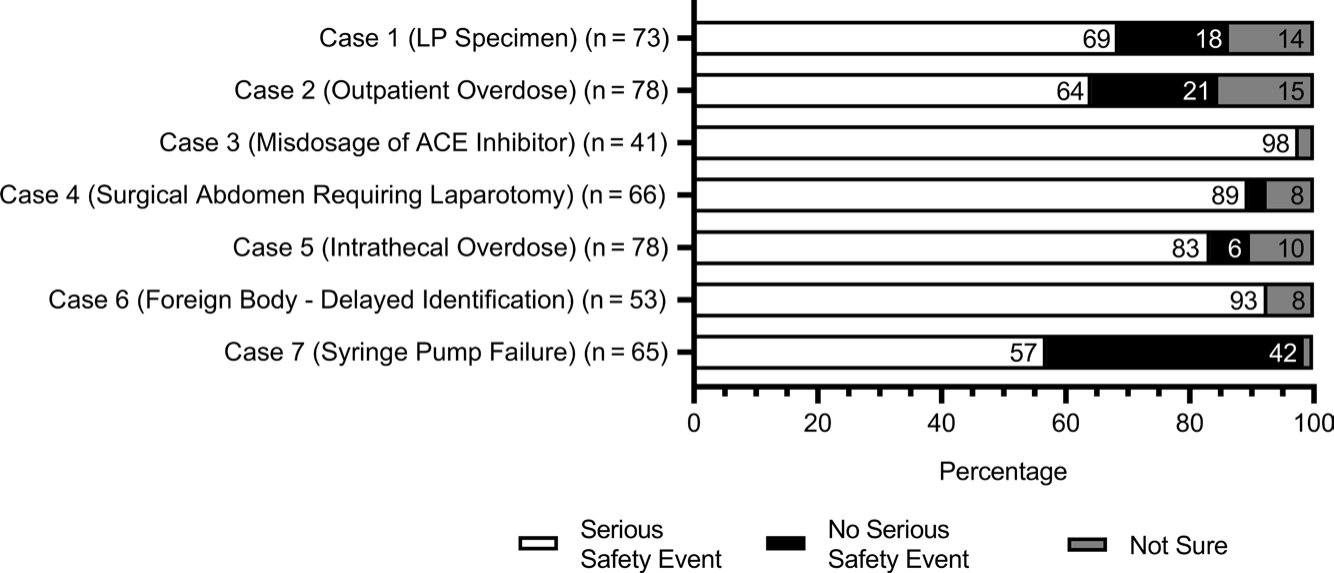
Serious safety event classification of vignettes.

Respondents who identified a deviation from GAPS in a vignette were then presented with a question to assess whether the resultant harm qualified as an SSE (Fig. 2). This survey portion included vignettes 2 and 5 (medication overdoses). The number of responses varied for each vignette as the survey questions were optional. The maximum number of possible responses was those who selected yes; a deviation occurred in the vignette from the previous survey question. The results revealed substantial or almost perfect levels of agreement among responses in vignettes 3 (misdosage of ACE inhibitor) (Gwet’s AC = 0.95), 4 (surgical abdomen) (Gwet’s AC = 0.78), 5 (intrathecal medication overdose) (Gwet’s AC = 0.66) and 6 (foreign body) (Gwet’s AC = 0.83) with at least 83% of respondents in each case classifying the event as an SSE (n = 40 of 41, n = 59 of 66, n = 65 of 78, and n = 49 of 53, respectively). Vignettes 1 (LP specimen), 2 (outpatient medication overdose), and 7 (syringe pump failure) demonstrated fair agreement in classification as an SSE (Gwet’s AC = 0.36, 0.28, and 0.32, respectively). In vignette 1, 69% classified the event as an SSE (n = 50 of 73), 18% did not (n = 13 of 73), and 14% were unsure (n = 10 of 73). Responses to vignette 2 were similar: 64% determined the event to be an SSE (n = 50 of 78), 21% determined it was not an SSE (n = 16 of 78), and 15% were unsure (n = 12 of 78). The respondents were more split in vignette 7, with 57% classifying the event as an SSE (n = 37 of 65), 42% stating it was not an SSE (n = 27 of 65), and 2% expressing uncertainty (n = 1 of 65).

## DISCUSSION

Variation exists among the hospitals that responded to our survey in all areas assessed: the presence of a deviation from GAPS, classification of vignettes with deviations and harm as SSEs, and SSE taxonomy utilized.

The first step in evaluating events in the SEC is determining whether there was a deviation from GAPS. Determining a deviation from GAPS requires several considerations, including the standards of care from which there may have been a deviation and if the adverse event resulted from a known complication.^23^ HPI states that “distinguishing between known complications and safety events, however, can be challenging” and developed a Known Complications Test to address this.^23^ Although this test may be helpful, many questions must be answered, and judgments need to be made to declare that a deviation has occurred. Given the many opportunities for and types of deviations in healthcare, it is simply impossible to define an objective standard of care to determine deviation in them all.

Forty percent of vignettes (n = 2 of 5) had a slight or fair agreement that a deviation occurred, and 60% (n = 3 of 5) had a substantial or near-perfect agreement. Four of five vignettes met our anticipated level of agreement, and one did not. Three of the vignettes that we anticipated to have variability in agreement regarding deviation from GAPS all involved provider practice concerns. Although some provider practice concerns are clear deviations, such as clear medication overdoses, many require review by provider peers to assess if a deviation from GAPS occurred. This peer review process usually occurs external to the SSE review team process, and the details are necessarily confidential. Thus, the determination of deviation in provider practice cases is likely to have variability based on the peer review processes at individual hospitals and the respondents’ experience with including peer review cases in their hospital’s SSE review process. For example, vignette 4 (surgical abdomen) had a substantial agreement in deviation compared with our anticipated variability in agreement. This finding may be due to a perceived clear deviation of the missing physical examination. Although the variation observed in responses to these illustrative vignettes may reflect a need for more information or special expertise to declare a deviation by respondents, two of the three cases were consistent with our expectation that respondents would have a variable agreement in the determination of a deviation from GAPS. We believe this variation reflects insufficient inter-rater reliability in applying HPI criteria across different healthcare organizations.

Next, SSE teams determine if the harm meets SSE criteria for events identified as having a deviation from GAPS. Fifty-seven percent (4 of 7) of the vignettes had a near-perfect agreement that an SSE occurred, and 43% (3 of 7) had a fair agreement that an SSE occurred. Four of the 7 vignettes met our anticipated level of agreement for SSE classification, and 3 of 7 did not. The three vignettes with only fair agreement were cases with lower severities of harm. HPI states that a factor in the variation of SSE capture is “inter-rater variation in classification of safety events with outcomes that fall at the border of moderate to minimal harm.”^23^ The determination of the severity of harm can be a controversial discussion point among safety event review teams. This controversy may be due to the absence of a standard definition utilized in the patient safety discipline for harm or classification of harm severity. It could further impact the variability in SSE determination across hospitals.^2,11–13^

While most (82% (n = 67)) hospitals are using the HPI SSE 1-5 taxonomy for evaluation and classification of adverse events as SSEs, 18% (n = 15) of respondent hospitals are utilizing a different taxonomy. Although hospitals utilizing the “Ohio modification” SSE 1–4 taxonomy were seeking to determine temporary harm more clearly and more consistently, it is likely that interpretive variation still exists among hospitals using any scoring system, as determining the level of harm requires discussion and judgment.^1,2,10–12^ HPI’s SEC attempts to mitigate this issue by providing examples of harm for each severity level and case examples for hospitals to utilize when applying the harm severity scoring. However, given the wide variety of safety events that occur in healthcare, teams applying the SSE classification to adverse events often need to make this determination without additional guidance.

Vignettes have been used in various ways in healthcare, including evaluating quality and safety, most notably to assess the agreement in identifying and classifying healthcare-associated infections (HAI) among various practitioners.^30–35^ Several studies of HAIs assessed the level of agreement by study participants to a correct answer determined by expert assessment.^31,33–35^ Several concluded the vignettes were useful to evaluate agreement among participants while reporting variability in the level of agreement, indicating the need for more training, experience, or definition improvement.^30–35^ They also concluded that the use of HAI rates for comparison of quality and safety across institutions is problematic based on the variation in agreement found.^30-34^ We are unaware of any studies evaluating the agreement in classifying SSEs. However, we believe the results of these HAI studies support the use of vignettes for this type of assessment and are consistent with the variation in the agreement of SSE classification we found.

There are several limitations to this study. First, the vignettes were brief. As a result, they did not have the fullness of a comprehensive case assessment typically available when an event occurs at a specific hospital. Second, hospitals also did not have the opportunity to ask clarifying questions that would be part of a true assessment process in a hospital’s event review system. These limitations may have impacted the respondent’s ability to determine if a deviation occurred and the severity of harm for SSE classification according to their respective SSE taxonomy and likely contributed to the proportions of unsure responses.

Additionally, single individuals or groups may have completed the surveys within hospitals and may not accurately reflect a hospital’s actual case adjudication in a comparable true case classification situation. SSE review processes may influence SSE case determinations; however, upon assessment, there were no characteristics of the respondents’ SSE review processes associated with the three questions posed for the vignettes (data not presented). Finally, all respondents were from children’s hospitals (either freestanding or within larger adult systems). Applying the SEC to pediatric patients may differ compared to adult-oriented programs.

Results demonstrated agreement and variability in determining deviation and SSE classification in the seven vignettes. The variation between hospitals in their interpretation and use of the HPI SEC demonstrates that comparing SSERs between organizations is problematic. It also highlights the importance of each hospital maintaining internal consistency over time in their team’s approach to case assessment and use of the SSE classification system. Changes in rates within a single hospital may reflect changes in the effectiveness of that organization’s safety efforts. HPI advocates that the SSER should be used only for directional benchmarking, with all seeking a downward direction of the SSER.^23^ Much effort, resources, and funds are dedicated to patient safety efforts and have not yet resulted in a reliable holistic measure to assess improvements. Just as we continuously improve the care that we deliver, we should work to continuously improve the metrics that we use to evaluate ourselves. It seems apparent that there is an opportunity for networks to improve inter-rater variability regarding both determinations of deviations from GAPS and the level of harm severity provided in the original HPI SEC.

Although the SSE methodology and metric used by participant pediatric hospitals yields generally similar review results, one must be cautious in using the SSER to compare patient safety outcomes across different hospitals.

## ACKNOWLEDGMENTS

The authors created the survey with input from patient safety team members at Children’s Hospital Colorado and the Clinical Steering Team members from SPS. Five respondents from different institutions also field-tested an initial version before the survey was finalized and disseminated.
